# Take Care of the Children

**Published:** 1857-09

**Authors:** 


### TAKE CARE OF THE CHILDREN.

On the fifth of February, 1854, two ladies of New York were conversing about the great suffering among the poor. One of them, *Mrs. Thomas Addis Emmett*, who had long been a manager of the *Marion Street* Lying-in Asylum, spoke of the miseries of infants, of their neglect, and the suffering of mothers whose poverty forced them to give the nourishment intended for their own infants, to the children of the rich. She told the following story: "A well-known sick-nurse, *Miss Sarah Richards*, called the evening before to see a lady whom she had attended, and while looking at the infant, she observed its wet-nurse was in tears. Miss Richards expressed her surprise that she should manifest such unhappiness while surrounded with every comfort. The poor nurse replied: 'It's just that makes me cry; for see what a nice bed, and good meals, and comfortable fire *I* have, while my own dear child may be starving or freezing, for I have promised the lady whose child I am nursing, never to nurse my own while I am nursing hers, and mine must be nursed by some one else.' On hearing this, Miss Richards, with a true woman's heart, sought out the child, and at night time, in a dirty basement room, found a sick woman lying on a miserable bed, who on being asked for the 'baby,' said, 'my baby died yesterday of the small-pox.'

"'But where is the nurse's baby?'

"'Oh! if that's what you want, here it is,' as she leaned over and drew from under her bed, a basket of soiled clothes, among which lay the child, whose mother might well weep for its utter wretchedness, neglect, and danger. The visitor stripped every rag from its little body, wrapped it in her shawl, took it to her own home, bathed and dressed it, sent for a physician, had it vaccinated that same night, and it was saved."*

Within a month from that night, ten thousand dollars were subscribed by generous citizens, a charter was obtained, and a society was organized for the purpose of taking care of the children of poor women; a home was procured; the whole thing went into immediate operation, and now, within three years, a building is in the course of erection, which alone will cost twenty-five thousand dollars. Since that memorable night, 553 persons have been taken in and cared for,—of these only thirty-six were American born. Of 160 children received, all but twenty were in a diseased state from pure neglect, such as burns, bruises, falls, or drugging. Some of these poor little creatures were frost-bitten. Yet, all but forty were saved, while in some of the European institutions of a similar character, according to the usual mortality, a hundred and twenty-eight would have died, instead of twenty; only thirty-two would have been saved instead of one hundred and forty.

Such are the ameliorations of disease, and such the life-saving results which spring up from sound medical views as to the best method of aiding the poor, the friendless, and forsaken of our race; such the results of woman's heart and the true physician's head, working together, thus preventing disease, and suffering, and death, rearing roses on the thorn bush, and planting flowers in the desert. To be co-workers with such, let every good citizen have a high ambition, and make a prompt, liberal, and cheerful donation to the NURSERY AND CHILD'S HOSPITAL of New York, conceived in woman's kindness, founded by woman's fair hands, and advocated by the eloquence of one of our own noblest senators.

---

*Sunday School Scholar.* Are all them old fellows living yet?

*Teacher.* Who, my child?

*Scholar.* Why, Abram, and Moses, and Deuteronomy, and all them.

---

* See Address of Hon ERASTUS BROOKS, at the laying of the corner stone of the *Nursery and Child's Hospital*, in New York, Lexington Avenue, *June 22d*, 1857, by *Mrs Cornelius Dubois*.

